# Maternal mortality: near-miss events in middle-income countries, a systematic review

**DOI:** 10.2471/BLT.21.285945

**Published:** 2021-08-30

**Authors:** Anke Heitkamp, Anne Meulenbroek, Jos van Roosmalen, Stefan Gebhardt, Linda Vollmer, Johanna I de Vries, Gerhard Theron, Thomas van den Akker

**Affiliations:** a Department of Obstetrics and Gynaecology, Stellenbosch University and Tygerberg Academic Hospital, Francie Van Zijl Avenue, Cape Town, 7505, South Africa.; b Department of Obstetrics and Gynaecology, Leiden University Medical Centre, Leiden, Netherlands.; c Athena Institute, Vrije Universiteit Amsterdam, Amsterdam, Netherlands.; d Department of Obstetrics and Gynaecology, Amsterdam UMC Vrije Universiteit Amsterdam, Amsterdam, Netherlands.

## Abstract

**Objective:**

To describe the incidence and main causes of maternal near-miss events in middle-income countries using the World Health Organization’s (WHO) maternal near-miss tool and to evaluate its applicability in these settings.

**Methods:**

We did a systematic review of studies on maternal near misses in middle-income countries published over 2009–2020. We extracted data on number of live births, number of maternal near misses, major causes of maternal near miss and most frequent organ dysfunction. We extracted, or calculated, the maternal near-miss ratio, maternal mortality ratio and mortality index. We also noted descriptions of researchers’ experiences and modifications of the WHO tool for local use.

**Findings:**

We included 69 studies from 26 countries (12 lower-middle- and 14 upper-middle-income countries). Studies reported a total of 50 552 maternal near misses out of 10 450 482 live births. Median number of cases of maternal near miss per 1000 live births was 15.9 (interquartile range, IQR: 8.9–34.7) in lower-middle- and 7.8 (IQR: 5.0–9.6) in upper-middle-income countries, with considerable variation between and within countries. The most frequent causes of near miss were obstetric haemorrhage in 19/40 studies in lower-middle-income countries and hypertensive disorders in 15/29 studies in upper-middle-income countries. Around half the studies recommended adaptations to the laboratory and management criteria to avoid underestimation of cases of near miss, as well as clearer guidance to avoid different interpretations of the tool.

**Conclusion:**

In several countries, adaptations of the WHO near-miss tool to the local context were suggested, possibly hampering international comparisons, but facilitating locally relevant audits to learn lessons.

## Résumé

**Objectif:**

Déterminer le taux d'incidence et les principales causes à l'origine des décès maternels évités de justesse dans les pays à moyen revenu en utilisant l'outil mis au point par l'Organisation mondiale de la Santé (OMS), et évaluer son applicabilité dans ce contexte.

**Méthodes:**

Nous avons procédé à une revue systématique des études portant sur les décès maternels évités de justesse dans les pays à moyen revenu, et publiées entre 2009 et 2020. Nous avons extrait des données relatives au nombre de naissances vivantes, au nombre de décès maternels évités de justesse, aux causes majeures qui les provoquent et aux dysfonctionnements organiques les plus fréquents. Nous avons également prélevé ou calculé le taux de décès maternels évités de justesse, le taux de mortalité maternelle et l'indice de mortalité. Enfin, nous avons retenu les descriptions des chercheurs concernant leurs expériences et adaptations de l'outil OMS au contexte local.

**Résultats:**

Nous avons inclus 69 études provenant de 26 pays (12 pays à revenu moyen inférieur, et 14 à revenu moyen supérieur). Ces études ont comptabilisé un total de 50 552 décès maternels évités de justesse sur 10 450 482 naissances vivantes. Le nombre médian de décès maternels évités de justesse par 1000 naissances vivantes s'élevait à 15,9 (écart interquartile, EI: 8,9–34,7) dans les pays à revenu moyen inférieur, et à 7,8 (EI: 5,0–9,6) dans les pays à revenu moyen supérieur, avec des variations considérables entre pays et au sein d'un même pays. Les causes les plus fréquentes à l'origine des décès évités de justesse étaient les hémorragies obstétricales (19 études sur 40 dans les pays à revenu moyen inférieur) et les problèmes d'hypertension (15 études sur 29 dans les pays à revenu moyen supérieur). Près de la moitié des études recommandait une adaptation aux critères de gestion et de laboratoire afin de ne pas sous-estimer le nombre de décès évités de justesse, ainsi que des orientations plus claires pour pouvoir interpréter l'outil sans équivoque.

**Conclusion:**

De nombreux pays ont suggéré d'adapter l'outil OMS au contexte local. Cela pourrait entraver les comparaisons internationales, mais faciliterait les audits localement pertinents permettant de tirer des enseignements.

## Resumen

**Objetivo:**

Describir la incidencia y las principales causas de los casos de morbilidad materna extrema en los países de ingresos medios mediante la herramienta correspondiente de la Organización Mundial de la Salud (OMS) y evaluar su aplicabilidad en estos contextos.

**Métodos:**

Se realizó una revisión sistemática de los estudios sobre morbilidad materna extrema en los países de ingresos medios que fueron publicados entre 2009 y 2020. Se obtuvieron datos sobre el número de nacidos vivos, el número de los casos de morbilidad materna extrema, las principales causas de los casos de morbilidad materna extrema y las disfunciones de órganos más frecuentes. Se extrajo, o se calculó, la tasa de morbilidad materna extrema, la tasa de mortalidad materna y el índice de mortalidad. Asimismo, se anotaron las descripciones de las experiencias de los investigadores y las modificaciones de la herramienta de la OMS para su uso local.

**Resultados:**

Se incluyeron 69 estudios de 26 países (12 de ingresos medios-bajos y 14 de ingresos medios-altos). Los estudios informaron de un total de 50 552 casos de morbilidad materna extrema por cada 10 450 482 nacidos vivos. La mediana del número de casos de morbilidad materna extrema por cada 1000 nacidos vivos fue de 15,9 (recorrido intercuartílico, IQR: entre 8,9 y 34,7) en los países de ingresos medios-bajos y de 7,8 (IQR: entre 5,0 y 9,6) en los países de ingresos medios-altos, con una variación considerable entre los países y dentro de ellos. Las causas más frecuentes de la morbilidad materna extrema fueron la hemorragia obstétrica en 19 de los 40 estudios de los países de ingresos medios-bajos y los trastornos hipertensivos en 15 de los 29 estudios de los países de ingresos medios-altos. Alrededor de la mitad de los estudios recomendaron adaptaciones de los criterios de laboratorio y de tratamiento para evitar la subestimación de los casos de morbilidad materna extrema, así como una orientación más clara para evitar las diferentes interpretaciones de la herramienta.

**Conclusión:**

En varios países se sugieren adaptaciones de la herramienta de la OMS sobre la morbilidad extrema a los contextos locales, lo que posiblemente dificulte las comparaciones internacionales, pero facilite la ejecución de auditorías pertinentes a nivel local para las lecciones aprendidas.

## ملخص

**الغرض:**

وصف الحدث والأسباب الأساسية للحالات وشيكة الوفاة للأمهات في الدول متوسطة الدخل، باستخدام أداة منظمة الصحة العالمية للحالات وشيكة الوفاة للأمهات، ولتقييم قابليتها للتطبيق في هذه الأوضاع.

**الطريقة:**

قمنا بمراجعة منهجية للدراسات حول الحالات وشيكة الوفاة للأمهات في الدول ذات الدخل المتوسط والتي نُشرت خلال الفترة من 2009 إلى 2020. قمنا باستخراج بيانات عن عدد المواليد الأحياء، وعدد الحالات وشيكة الوفاة للأمهات، والأسباب الرئيسية للحالات وشيكة الوفاة للأمهات، والخلل الوظيفي الأكثر شيوعًا في الأعضاء. قمنا باستخراج أو حساب نسبة الحالات وشيكة الوفاة للأمهات، ونسبة وفيات الأمهات، ومؤشر الوفيات. ولاحظنا أيضًا توصيفات تجارب الباحثين، وتعديلات أداة منظمة الصحة العالمية للاستخدام المحلي.

**النتائج:**

قمنا بتضمين 69 دراسة من 26 دولة (12 دولة من الشريحة الدنيا للدول ذات الدخل المتوسط، و14 دولة من الشريحة العليا للدول ذات الدخل المتوسط). أوضحت الدراسات وجود إجمالي 50552 من الحالات وشيكة الوفاة للأمهات من أصل 10450482 حالة للمواليد الأحياء. كان متوسط عدد الحالات وشيكة الوفاة للأمهات لكل 1000 من المواليد الأحياء هو 15.9 (المدى بين الشرائح الربعية (IQR): 8.9 إلى 34.7) في الدول من الشريحة الدنيا للدول ذات الدخل المتوسط (المدى بين الشرائح الربعية: 5.0 إلى 9.6) في الدول من الشريحة العليا للدول ذات الدخل المتوسط، مع تباين ملموس بين الدول وداخلها. كانت الأسباب الأكثر شيوعًا للحالات وشيكة الوفاة هي النزف أثناء الولادة في 19/40 دراسة في الدول من الشريحة الدنيا للدول ذات الدخل المتوسط، واضطرابات ارتفاع ضغط الدم في 15/29 دراسة في الدول من الشريحة العليا للدول ذات الدخل المتوسط. أوصى ما يقرب من نصف الدراسات بإجراء تعديلات على معايير المختبر والإدارة لتجنب التقليل من شأن أية حالة وشيكة الوفاة، فضلًا عن إرشادات أوضح لتجنب التفسيرات المختلفة للأداة.

ا **لاستنتاج ** في العديد من الدول، تم اقتراح تعديلات على أداة منظمة الصحة العالمية للحالات وشيكة الوفاة لتلائم السياق المحلي، مما قد يعيق المقارنات الدولية، ولكنه يسهل عمليات التدقيق المحلية ذات الصلة للاستفادة من الدروس.

## 摘要

**目的:**

使用世界卫生组织 (WHO) 的孕产妇险兆事件工具描述中等收入国家孕产妇险兆事件的发生率和主要原因,并评估该工具在这些事件中的适用性。

**方法:**

我们对 2009 2020 年发表的关于中等收入国家孕产妇险兆事件研究进行系统地回顾。提取了关于活产婴儿数量、孕产妇险兆事件数量、孕产妇险兆事件主要原因以及最常见的器官功能障碍数据。我们提取或计算出孕产妇险兆率、孕产妇死亡率和死亡率指数。我们还注意到对研究人员经验的描述以及对世卫组织工具进行的本地适应性改进。

**结果:**

我们纳入了来自 26 个国家(12 个中低收入国家和 14 个中高收入国家)的 69 项研究。研究报告了 10,450,482 例活产儿中共有 50,522 起孕产妇险兆事件。中低收入国家每 1000 例活产儿中孕产妇险兆事件的中位数为 15.9(四分位差,IQR:8.9-34.7),中高收入国家中位数为 7.8 (IQR: 5.0-9.6),国家之间和国家内部都存在巨大的差异。最常见的险兆事件原因,中低收入国家 19/40 的研究显示为产科出血,中高收入国家 15/29 的研究显示为高血压疾病。已建议对大约一半的研究进行实验室和管理标准调整,以及更清晰的指导,以避免过低估计险兆事件和对该工具的解释产生歧义。

**结论:**

在一些国家,已建议根据当地情况调整世卫组织险兆事件工具,这可能会妨碍国际对比,但有助于当地开展相关审查以总结经验教训。

## Резюме

**Цель:**

Описать частоту возникновения случаев и основные причины осложнений при родах, представляющих угрозу для жизни матери, в странах со средним уровнем дохода с помощью инструмента Всемирной организации здравоохранения (ВОЗ) для оценки такого рода осложнений, а также оценить его применимость в данных условиях.

**Методы:**

Был выполнен систематический обзор исследований, посвященных осложнениям при родах, представляющим угрозу для жизни матери, в странах со средним уровнем дохода, которые были опубликованы в 2009–2020 гг. Авторы извлекли данные о количестве живорождений, количестве случаев таких осложнений, основных причинах таких осложнений и наиболее частой дисфункции органов. Авторы также извлекли или рассчитали коэффициент осложнений, коэффициент материнской смертности и индекс смертности. Авторы также обратили внимание на описание опыта исследователей и модификации инструмента ВОЗ для использования на местах.

**Результаты:**

В обзор было включено 69 исследований из 26 стран (12 стран с уровнем дохода ниже среднего и 14 стран с уровнем дохода выше среднего). Из 10 450 482 живорождений исследования сообщили в общей сложности о 50 552 случаях осложнений при родах, представляющих угрозу для жизни матери. Среднее число случаев таких осложнений на 1000 живорождений составило 15,9 (межквартильный размах, МКР: 8,9–34,7) в странах с доходом ниже среднего и 7,8 (МКР: 5,0–9,6) в странах с доходом выше среднего со значительными различиями между странами и внутри стран. Наиболее частыми причинами осложнений были акушерские кровотечения в 19 из 40 исследований в странах с уровнем дохода ниже среднего и гипертонические нарушения в 15 из 29 исследований в странах с уровнем дохода выше среднего. Около половины исследований рекомендовали адаптацию к лабораторным и управленческим критериям во избежание недооценки случаев такого рода осложнений, а также более четкое руководство во избежание различных интерпретаций инструмента.

**Вывод:**

Для извлечения уроков в нескольких странах была предложена адаптация инструмента ВОЗ для оценки осложнений при родах, представляющих угрозу для жизни матери, к местным условиям, что, возможно, затруднило бы международные сопоставления, но облегчило бы проведение актуального аудита и извлечение опыта на местном уровне.

## Introduction

Women are at risk of developing severe morbidity and mortality during pregnancy, childbirth and postpartum, especially in low-income and middle-income countries where 99% of all maternal deaths occur.^1^ Improvement of maternal health is urgently needed and one of the sustainable development goals is to reduce the global maternal mortality ratio to less than 70 per 100 000 live births by 2030.^2^

In addition to maternal mortality, severe maternal morbidity is used as an indicator of quality of maternity care.^3^^,^^4^ Measuring and comparing outcomes of severe maternal morbidity studies have been difficult because of the use of different identification criteria.^5^^,^^6^ In 2009, the World Health Organization (WHO) developed the maternal near-miss tool to introduce a universal approach to comparing the quality of maternity care between different countries.^6^^–^^10^ Maternal near miss is defined by WHO as “a woman who nearly died but survived a complication that occurred during pregnancy, childbirth or within 42 days of termination of pregnancy.”^6^^–^^10^ Maternal near miss occurs more frequently than maternal death and by evaluating the condition, more robust lessons may be learnt about quality of care.^5^^,^^6^

Several studies, however, have demonstrated difficulties in applying the tool.^11^^–^^13^ Box 1 shows the WHO maternal near-miss criteria for determining life-threatening conditions and additional criteria for baseline assessment of quality of care. Among the requirements to meet the various criteria of the tool are: advanced laboratory diagnostic tests; large numbers of units of blood in transfusion as the threshold to identify severe haemorrhage; and intensive clinical monitoring. Some of these requirements cannot easily be met in low-resource settings due to limited diagnostic capacity and reduced options for medical intervention in these settings, which may lead to underestimation of the incidence of maternal near miss.^13^ Researchers in sub-Saharan Africa have suggested adaptations of the maternal near-miss tool for use in low-income countries.^14^^,^^15^ But even in high-income countries, where sufficient resources should be available, there has been discussion about what the appropriate inclusion criteria for maternal near miss should be.^16^ Identification of maternal near miss was found to be compromised by incomplete documentation in the medical records to establish whether maternal near-miss criteria were met.

Box 1 **Inclusion criteria in the WHO near-miss approach for maternal health**

**Life-threatening conditions (near-miss criteria)**

- Cardiovascular dysfunction: shock; cardiac arrest (absence of pulse or heartbeat and loss of consciousness); use of continuous vasoactive drugs; cardiopulmonary resuscitation; severe hypoperfusion (lactate > 5 mmol/L or > 45 mg/dL); severe acidosis (pH < 7.1)
- Respiratory dysfunction: acute cyanosis; gasping; severe tachypnoea (respiratory rate > 40 breaths per minute); severe bradypnoea (respiratory rate < 6 breaths per minute); intubation and ventilation not related to anaesthesia; severe hypoxaemia (oxygen saturation < 90% for ≥ 60 minutes or PaO_2_/FiO_2_ < 200)
- Renal dysfunction: oliguria non-responsive to fluids or diuretics; dialysis for acute renal failure; severe acute azotaemia (creatinine ≥ 300 μmol/mL or ≥ 3.5 mg/dL)
- Coagulation or haematological dysfunction: failure to form clots; massive transfusion of blood or red cells (≥ 5 units of blood); severe acute thrombocytopenia (< 50 000 platelets/mL)
- Hepatic dysfunction: jaundice in the presence of pre-eclampsia; severe acute hyperbilirubinaemia (bilirubin > 100 μmol/L or > 6.0 mg/dL)
- Neurological dysfunction: prolonged unconsciousness (lasting ≥ 12 hours) or coma (including metabolic coma); stroke; uncontrollable fits or status epilepticus; total paralysis
- Uterine dysfunction: uterine haemorrhage or infection leading to hysterectomy

**Severe maternal complications (additional categories for baseline assessment of quality of care)**

- Severe postpartum haemorrhage
- Severe pre-eclampsia
- Eclampsia
- Sepsis or severe systemic infection
- Ruptured uterus
- Severe complications of abortion

**Critical interventions or intensive care unit use (additional categories for baseline assessment of quality of care)**

- Admission to intensive care unit
- Interventional radiology
- Laparotomy (includes hysterectomy; excludes caesarean section)
- Use of blood products

PaO_2_/FiO_2_: ratio of arterial oxygen partial pressure to fractional inspired oxygen; WHO: World Health Organization.Source: WHO, 2011.^10^

Reports about the incidence of maternal near miss have been published for several high- and low-income countries, and the applicability of the WHO maternal near-miss tool has been evaluated in several of these. However, data are lacking about maternal near miss in middle-income countries. We therefore made a systematic review of the incidence and main causes of maternal near miss in middle-income countries. We also aimed to evaluate qualitative findings documented by researchers with regard to applicability of the tool and suggest possible adaptations of the WHO maternal near-miss approach for middle-income settings.

## Methods

We conducted the review according to the Preferred Reporting Items for Systematic Reviews and Meta-Analyses guideline,^17^ and registered with the International Prospective Register of Systematic Reviews (CRD42021232735). 

### Study selection

We performed a search of online databases for articles on maternal near miss in middle-income countries published between 1 January 2009 and 12 November 2020 without language restrictions. The earlier date was chosen since 2009 is the year when the WHO maternal near-miss approach was first published.^6^^,^^9^ Retrospective studies that used data from before 2009 were included only if they made use of the WHO definition for maternal near miss. 

We used the keywords “severe acute maternal morbidity,” “maternal near miss” and “middle income country.” Since PubMed® does not provide medical subject headings terms for country income groups, we first determined which countries were classified as middle-income and inserted each country name as a separate term in the search strategy. The search was last run in November 2020 in the online databases PubMed®, Embase®, Web of Science, Cochrane Library, Emcare and Academic Search Premier. In addition, we searched the following regional databases: Index Medicus for the Eastern Mediterranean Region; Index Medicus for South-East Asia Region, Latin America and the Caribbean; African Index Medicus; IndMED; and Global Health Library. More details of the search strategy are in the data repository.^18^

We included studies that met all four inclusion criteria: (i) articles about maternal near miss as defined by WHO; (ii) data on the incidence of maternal near miss per 1000 live births and the main causes; (iii) describing countries meeting the World Bank classification for middle income;^19^^,^^20^ and (iv) reporting the specific criteria used to identify maternal near miss and experiences with applying the WHO maternal near-miss criteria, including possible modifications of the WHO maternal near-miss tool for local use. We included studies containing multiple countries only if outcomes per country could not be found elsewhere. If multiple studies published data on the same country, all of them were reviewed and included. We used the World Bank classifications by gross national income per capita to determine country income groups.^19^^,^^20^ As the classification of several countries changed over the search dates, we included studies if countries were middle-income in the year of publication, as classified by the World Bank at that time.

We excluded studies that: (i) did not apply WHO maternal near miss definitions; (ii) only focused on one specific disease or risk factor without providing overall data on maternal near miss; (iii) were comments, abstracts, secondary analysis or surveys of existing studies; (iv) only focused on neonatal outcomes; or (v) only described the process of health care or methods of identifying maternal near miss without providing incidence or most frequent causes, and without providing qualitative findings with regard to applicability and adaptations of the tool.

Two independent researchers screened all citations initially for relevance based on title and abstract and selected studies for inclusion after reading the full-text papers. Disagreements were resolved in a discussion between these two reviewers to reach a consensus. In case no consensus could be reached, the reviewers consulted a third researcher to reach an agreement on inclusion of articles. 

### Data extraction 

We extracted data on the number of live births, number of cases of maternal near miss and number of maternal deaths. Where available, we noted the following indicators: maternal near-miss ratio (number of cases of maternal near miss per 1000 live births), maternal mortality ratio (number of maternal deaths per 100 000 live births), ratio of maternal near miss to maternal death (number of cases of maternal near miss ÷ the number of maternal deaths) and mortality index [number of maternal deaths ÷ (number of cases of maternal near miss + number of maternal deaths) × 100]. If indicators were missing for any study, we calculated the values from the available data. We also extracted data on the most frequent organ dysfunction and the most frequent cause of maternal near miss. When studies included qualitative comments on the methods of using the WHO maternal near-miss approach, we noted any modifications to the WHO tool applied in the studies and any problems reported by the study researchers. When articles described the use of multiple methods to identify maternal near miss, we only reported data concerning use of the WHO maternal near-miss tool. 

### Data analysis 

We subdivided the countries for analysis into lower-middle income and upper-middle income according to the World Bank categories.^19^^,^^20^ We report the number of studies and the frequency of causes of near miss as numbers and percentages. We calculated the median values and interquartile range (IQR) of the maternal indicators if the data were not normally distributed. We performed statistical analysis using SPSS version 24.0 (IBM Corp., Armonk, United States of America). 

We estimated risk of bias in individual studies by quality assessment of studies. Studies were considered to be of acceptable quality if: (i) there was a clear description of the study population with a minimum of 100 live births over a period of at least 3 months; (ii) new cases of maternal near miss were identified in daily audits or rounds by trained medical staff; and (iii) the setting was an entire hospital rather than only one intensive care unit. The two reviewers who selected the studies did the quality assessment. We amended the Newcastle–Ottawa scale^21^ for this study by coding the item Selection of the non-exposed cohort as not applicable (NA). The maximum quality score was therefore 8 instead of the original score 9 in the Newcastle–Ottawa Scale; more details are in the data repository.^18^

### Ethical approval

Ethical approvals were obtained from the Health Research Ethics Committee (HREC), Faculty of Health Sciences, Stellenbosch University, on 3 October 2018 (Project ID: 1427, HREC Reference #: S18/02/023) and from the Provincial Health Authority, the chief executive officer of Tygerberg Hospital and the heads of respective departments. 

## Results

**Fig. 1:**
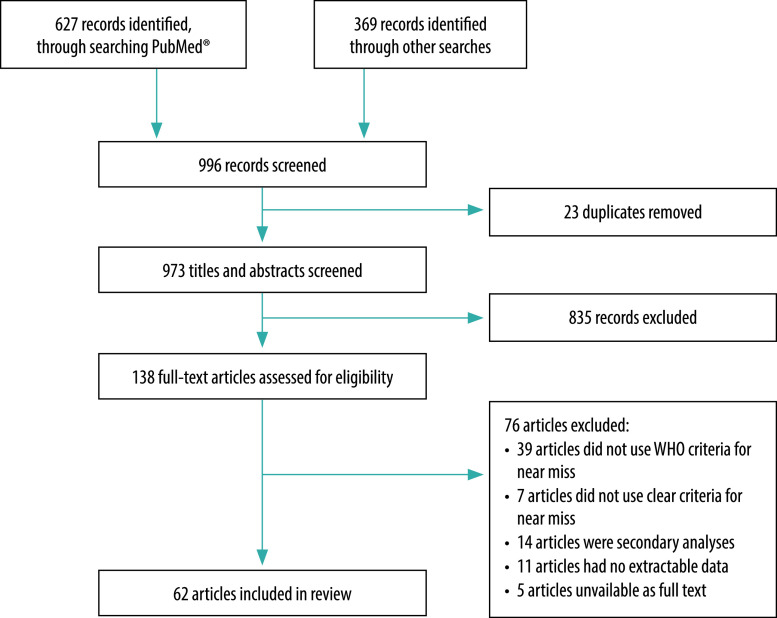
Flowchart of studies included in the systematic review of maternal near miss in middle-income countries

The search resulted in 996 records. After removal of duplicates, we screened 973 articles based on title and abstract, after which 138 articles were retrieved for full-text evaluation. Of these, we excluded 76 articles (39 of which did not apply the WHO maternal near-miss tool; Fig. 1). For the final review we included 62 articles.^22^^–^^83^ Our quality assessment of the articles showed the following scores: eight articles with score 4; 15 articles with score 5; 26 articles with score 6 and 13 articles with score 7. No articles described possible missing data in the follow-up period which resulted in none of the articles having a maximum score of 8.

The included articles reported data from 69 studies in 26 countries (12 lower-middle-income countries and 14 upper-middle-income countries). Two of the articles^30^^,^^83^ presented data on multiple countries. Of the 69 studies, 40 (58%) were done in lower-middle-income countries and 29 (42%) in upper-middle-income countries. Half (35 studies) of them, were conducted in one or more tertiary health-care facility. General descriptions of the studies and differences in methods are summarized in Table 1 (available at: https://www.who.int/publications/journals/bulletin/). Four retrospective studies described data from before 2009 using the WHO definition for maternal near miss.^23^^–^^26^

**Table 1:** Characteristics of studies included in the review on maternal near miss in middle-income countries

Author	Setting	Study period	Study type	Medical care setting	Primary objective	Data source	Identification of cases of maternal near miss done by	Training of staff	Follow-up of the patient after end of pregnancy
**Lower-middle-income countries**									
Ps et al., 2013^53^	India, Karnataka	2011–2012	Audit	1 tertiary referral hospital with 6 primary health centres attached	To determine incidence of maternal near miss	NR	NR	NR	42 days
Tunçalp et al., 2013^36^	Ghana	2010–2011	Prospective descriptive	1 tertiary referral centre	To assess incidence of maternal near miss and related indicators	Medical records	NR	NR	42 days
Kaur et al., 2014^43^	India, Himachal Pradesh	2012–2013	Prospective observational	1 tertiary care hospital	To assess the causes and incidence of maternal near miss	NR	NR	NR	42 days
Kushwah et al., 2014^48^	India, Madhya Pradesh	2012–2013	Prospective cross-sectional	1 government tertiary care referral centre	To describe profile and outcomes of maternal near miss	Daily identification of women with maternal near miss in wards	Investigator	NR	42 days
Luexay et al., 2014^61^	Lao People's Democratic Republic	2011	Descriptive prospective	243 villages (community and local hospitals)	To determine incidence and causes of maternal near miss and maternal death in Lao People's Democratic Republic	Daily home visits	Health volunteers and health-centre staff	Yes	42 days
Nacharajuh et al., 2014^55^	India	2012–2014	NR	1 rural medical college	To assess number of maternal near misses and maternal near miss ratio	NR	NR	NR	42 days
Pandey et al., 2014^42^	India	2011–2012	Retrospective	1 tertiary hospital	To assess frequency and nature of maternal near miss	Medical records	NR	NR	42 days
Bakshi et al., 2015^31^	India	NR	Cross-sectional epidemiological	2 primary, 1 community and 1 tertiary facility	To determine prevalence and indicators of maternal near miss	Medical records	NR	NR	42 days
Mazhar et al., 2015^66^	Pakistan	2011	Cross-sectional	16 government facilities	To determine incidence and causes of severe maternal outcome	Medical records	Coordinators and data collector	Yes	7 days
Sangeeta et al., 2015^27^	India	2012–2013	Prospective	1 tertiary referral centre	To determine frequency and analyse causes of complications of maternal near miss and deaths	Medical records	NR	NR	42 days
Abha et al., 2016^49^	India, Raipur	2013–2015	Prospective observational	1 medical college hospital	To audit maternal near miss and to review substandard care	Clinical examinations; laboratory results and criteria meeting the WHO maternal near-miss criteria	NR	NR	42 days
Ansari et al., 2016^65^	Pakistan	2013	Cross-sectional descriptive	Obstetric unit of 1 tertiary referral centre	To determine frequency and nature of maternal near miss	NR	NR	NR	42 days
Kulkarni et al., 2016^50^	India, Maharashtra	2012–2014	Prospective observational	2 tertiary centres	To investigate incidence and patterns of maternal near miss and to study classification criteria	Hospital registers; patient interviews	Research officers	No	42 days
Oladapo et al., 2016^62^	Nigeria	2012–2013	Cross-sectional	42 tertiary hospitals	To investigate burden and causes of life-threatening maternal complications and quality of obstetric care	Medical records collected during daily ward rounds	Trained data collector	Yes	42 days
Parmar et al., 2016^35^	India	2012	Cross-sectional	1 tertiary referral hospital	To describe incidence of maternal near miss	In-depth patient interviews	Investigators	NR	42 days
Rathod et al., 2016^51^	India, Maharashtra	2011–2013	Retrospective cohort	1 tertiary referral centre	To determine incidence of maternal near miss	Medical records	NR	NR	42 days
Ray et al., 2016^33^	India, Maharashtra	2014–2015	Cross-sectional observational	1 tertiary referral centre	To determine prevalence of maternal near miss	NR	NR	NR	42 days
Tanimia et al., 2016^40^	Papua New Guinea	2012–2013	Prospective observational	1 teaching referral hospital	To assess routinely collected data and determine rates of maternal near miss	Identification of women with maternal near miss in daily ward rounds and discussions in unit meetings	House officers	NR	NR
Bolnga et al., 2017^68^	Papua New Guinea	2014–2016	Prospective observational	1 provincial hospital	To determine maternal near-miss ratio, mortality index and associated indices	Identification of women with maternal near miss in wards	Obstetric team	NR	NR
Chandak & Kedar, 2017^52^	India, Maharashtra	2013–2015	Cross-sectional observational	1 tertiary care institute	To determine frequency and nature of maternal near miss	NR	NR	NR	42 days
Mbachu et al., 2017^28^	Nigeria	2014–2015	Cross-sectional	1 tertiary centre	To evaluate maternal near miss and maternal deaths	Medical records by daily rounds	Medical officer and interns	NR	42 days
Tallapureddy et al., 2017^56^	India	2014	Retrospective cohort	1 tertiary care hospital	To study severe maternal outcome and use WHO maternal near-miss tool	Admissions and medical records	NR	NR	42 days
Panda et al., 2018^32^	India, Odisha	2017	Cross-sectional	1 tertiary care hospital	To estimate burden of maternal near miss	Medical records	NR	NR	42 days
Reena & Radha, 2018^54^	India, Kerala	2011–2012	Cross-sectional	1 government medical college	To determine frequency, nature and timing of delays in cases of maternal near miss	Medical records; patient interviews	Obstetrician	NR	NR
Chaudhuri & Nath, 2019^57^	India, Kolkata	2013–2014	Prospective observational	1 tertiary care hospital	To test application of clinical definition of life-threatening complications in pregnancy and to determine the level of near-miss maternal morbidity and mortality due to life-threatening obstetric complications	Medical records	Doctors, nurses and investigator	No	42 days
Chhabra et al., 2019^58^	India, Delhi	2013–2014	Case–control	1 tertiary level	To study incidence of severe maternal morbidity and maternal near miss, to assess feasibility of application of criteria and to assess causes and associated factors	Daily ward visits; medical records	Investigator	No	42 days
El Agwany, 2019^46^	Egypt, Alexandria	2015–2016	Retrospective cohort	1 tertiary level	To assess characteristics of maternal near miss by applying WHO approach	Intensive care unit medical records	Investigators	NR	42 days
Gabbur et al., 2019^59^	India, Karnataka	2015–2017	Case series	1 tertiary level	To assess maternal near miss and responsible factors	Medical records	NR	NR	42 days
Herklots et al., 2019^69^	United Republic of Tanzania, Zanzibar	2017–2018	Prospective cohort	1 main referral hospital	To determine correlation between number of organ dysfunctions and risk of mortality and to calculate sensitivity and specificity	Medical records	Junior investigators and research assistants	Yes	42 days
Jayaratnam et al., 2019^71^	Timor-Leste	2015–2016	Prospective observational	Main referral hospital (only tertiary hospital in country)	To determine rate of severe maternal outcomes and most common etiologies	Daily ward rounds; medical records	Investigator and assistant investigators	NR	42 days
Mansuri & Mall, 2019^60^	India, Ahmedabad City	2015–2016	Cross-sectional study, facility-based retrospective	4 tertiary care centres	To describe the demographic characteristics of near miss patients and to determine the indicators of severe maternal morbidity and mortality	Second-day ward rounds; medical records	NR	NR	NR
Oppong et al., 2019^47^	Ghana	2015	Cross-sectional and case–control	3 tertiary referral hospitals	To explore incidence and factors associated with maternal near miss	Medical records	Research assistants	Yes	42 days
Karim et al., 2020^67^	Pakistan	2016−2017	Descriptive	Tertiary hospital	To describe types and frequencies of maternal near miss	Identification of cases during admission	NR	NR	42 days
Lilungulu et al., 2020^70^	United Republic of Tanzania, Dodoma	2015–2016	Retrospective	1 regional referral hospital	To identify magnitude and predictors of maternal and perinatal mortality among women with severe maternal outcome	Identification of cases during admission and in the wards	Three investigators	NR	NR
Owolabi et al., 2020^22^	Kenya	2018	Cross-sectional	16 county hospitals, 2 national level hospitals and 46 subcounty hospitals	To determine incidence and causes of maternal near miss	Identification of cases in wards; medical records; patient interviews in case of missing data	Identified ”study clinician” such as Medical officers and nurses	Yes	42 days
Samuels & Ocheke, 2020^64^	Nigeria	2012–2013	Cross-sectional	1 university hospital	To determine frequency of maternal near miss and maternal deaths to identify common causes	Identification of cases during admission and in the wards; medical records	NR	NR	42 days
Ugwu et al., 2020^63^	Nigeria	2013–2016	Prospective	1 hospital	To determine frequency of maternal near miss and maternal deaths, to document primary causative factor and to compare maternal near miss and maternal deaths	Medical records	Research assistants (residents in internal medicine)	Yes	42 days
**Upper-middle-income countries**									
Cecatti et al., 2011^24^	Brazil, São Paulo State	2002–2007	Retrospective	Intensive care unit of 1 tertiary referral centre	To evaluate WHO maternal near-miss criteria	Medical records	Investigators and research assistants	NR	42 days
Morse et al., 2011^23^	Brazil, Rio de Janeiro	2009	Cross-sectional prospective	1 regional public referral hospital	To investigate severe maternal morbidity and maternal near miss using different identification criteria	Medical records; identification of cases during daily ward rounds	Principal investigator and trained students	Yes	42 days
Lotufo et al., 2012^25^	Brazil, São Paulo State	2004–2007	Cross-sectional retrospective	Intensive care unit of 1 university referral hospital	To study maternal morbidity and mortality among women in intensive care	Medical records	Investigator	No	42 days
Jabir et al., 2013^82^	Iraq	2010	Cross-sectional	6 public hospitals	To use WHO maternal near-miss tool to assess characteristics and quality of care in women with severe complications	Medical records; daily staff interviews	Coordinators	Yes	7 days
Shen et al., 2013^26^	China	2008–2012	Retrospective	1 private tertiary hospital	To investigate factors associated with maternal near miss and mortality	Medical records	Audit committee of obstetricians and specialist registrars	Yes	42 days
Dias et al., 2014^72^	Brazil, nationwide	2011 – 2012	National, hospital-based study of women who have recently given birth and their newborns	1043 hospitals	To estimate incidence of maternal near miss in hospitals	Medical records; patient interviews	Students and health-care workers, coordinators from different health facilities and specialists	Yes	42 days
Galvão et al., 2014^74^	Brazil, Sergipe	2011–2012	Cross-sectional and case–control	2 reference maternity hospitals	To determine prevalence of severe acute maternal morbidity and maternal near miss and to identify risk factors	Identification of cases in wards; medical records; patient interviews	Obstetrician and trained staff	Yes	42 days
Madeiro et al., 2015^75^	Brazil, Piaui	2012–2013	Prospective	1 public tertiary referral hospital	To investigate incidence and determinants of severe maternal morbidity and maternal near miss	Medical records	Trained investigators	Yes	42 days
Naderi et al., 2015^80^	Islamic Republic of Iran	2013	Prospective	8 hospitals	To estimate incidence and identify underlying factors of severe maternal morbidity	Identification of cases during admission and in the wards	Midwife and gynaecologist	NR	42 days
Oliveira & Da Costa, 2015^76^	Brazil, Pernambuco	2007–2010	Descriptive cross-sectional	Obstetric intensive care unit of 1 tertiary hospital	To analyse epidemiological and clinical profile of maternal near miss	Medical records	Investigator and research assistants	Yes	42 days
Soma-Pillay et al., 2015^37^	South Africa	2013–2014	Descriptive population-based	9 delivery facilities	To determine spectrum of maternal morbidity and mortality	Medical records; daily audit meetings	NR	No	42 days
Cecatti et al., 2016^73^	Brazil, nationwide	2009 – 2010	Cross-sectional	27 referral maternity hospitals	To identify severe maternal morbidity cases, study their characteristics and test WHO criteria	Medical records	Medical coordinators	Yes	42 days
Ghazivakili et al., 2016^81^	Islamic Republic of Iran	2012	Cross-sectional	13 public and private hospitals	To assess incidence of maternal near miss and audit quality of care	Medical records	Midwives with data collection form	Yes	7 days
Mohammadi et al., 2016^39^	Islamic Republic of Iran	2012–2014	Incident case–control	3 university hospitals; 1 secondary, 2 tertiary	To determine frequency, causes, risk factors and perinatal outcomes of maternal near miss	Medical records	Investigators	NR	42 days
Norhayati et al., 2016^29^	Malaysia	2014	Cross-sectional	2 referral and tertiary hospitals	To study severe maternal morbidity and maternal near miss and related indicators	Hospital and home-based medical records	Research assistant trained in nursing	No	42 days
Akrawi et al., 2017^41^	Iraq	2013	Cross-sectional	1 maternity teaching hospital	To determine major determinants of maternal near miss and maternal death	Medical records; interviews of women who experienced maternal near miss	NR	NR	42 days
Iwuh et al., 2018^44^	South Africa	2014	Retrospective observational	3 hospitals (secondary and tertiary)	To measure maternal near-miss ratio, maternal mortality ratio and mortality index	Medical records	Investigator and health-care providers, with identification confirmed by senior obstetric specialists	No	42 days
Oliveira Neto et al., 2018^77^	Brazil, São Paulo State	2013 – 2015	Retrospective cross-sectional	Obstetric intensive care unit of 1 public teaching hospital	To explore indicators of WHO maternal near-miss criteria	Medical records	NR	NR	42 days
De Lima et al., 2019^34^	Brazil, Alagoas	2015–2016	Prospective cohort observational	1 tertiary	To collect data on maternal near miss	Patient interviews; medical records at admission and at day 42	Principle investigator and research assistants	NR	42 days
Mu et al., 2019^79^	China	2012–2017	Population-based surveillance system	461 health facilities	To introduce maternal near miss into a national surveillance system and to report maternal near miss	Medical records, web-based online reporting system	Obstetrician and nurses responsible for patient care	Yes	42 days
Heemelaar et al., 2020^38^	Namibia	2018–2019	Nationwide surveillance	All public hospitals (1 tertiary, 4 regional, 30 district)	To obtain data on pregnancy outcomes and assess benefits of such surveillance in comparison with surveillance of maternal deaths only	Medical records	Nominated staff	Yes	42 days
Ma et al., 2020^78^	China	2012–2018	Cross-sectional	18 hospitals in province	To explore prevalence of maternal near miss, risk factors for maternal near miss and relationship between maternal near miss and perinatal outcomes	Electronic medical record system	Nurses and doctors	Yes	42 days
Verschueren et al., 2020^45^	Suriname	2017–2018	Prospective nationwide population-based cohort	All 5 hospitals and primary health-care centre	To find reason for high maternal mortality ratio and stillbirths and compare findings with other countries to improve quality of care	Identification of cases during daily ward rounds; medical records	Research coordinator (doctor) and investigator	Yes	42 days
**Multiple countries**									
Bashour et al., 2015^83^	Egypt, Lebanon	2011	Cross-sectional	Public maternity hospitals	To report on prevalence of maternal near miss	Medical records	Investigators	Yes	7 days
De Mucio et al., 2016^30^	Colombia, Dominican Republic, Ecuador, Honduras, Nicaragua, Paraguay, Peru	2013	Cross-sectional	Hospitals multiple countries	To evaluate performance of a systematized form to detect severe maternal outcomes	Medical records	Health-care professionals	Yes	42 days

NR: data not reported; WHO: World Health Organization.

### Incidence

The incidence and causes of maternal near miss in middle-income countries are presented in Table 2. The studies reported a total of 50 552 maternal near misses out of the total live births of 10 450 482. Overall, the median maternal near-miss ratio in these middle-income countries was 9.6 per 1000 live births (IQR: 7.0–23.3). In lower-middle-income countries the median maternal near-miss ratio was 15.9 per 1000 live births (IQR: 8.9–34.7), ranging from 4.0 in an Indian government tertiary care centre^27^ to 198.0 in a private tertiary care centre in Nigeria.^28^ For upper-middle-income countries, the median maternal near-miss ratio was 7.8 per 1000 live births (IQR: 5.0–9.6), ranging from 2.2 in two Malaysian tertiary hospitals^29^ to 54.8 in Brazil.^34^

**Table 2:** Incidence and causes of maternal near miss in middle-income countries

Author	Setting	No. of live births	No. of cases of maternal near miss	Maternal near misses per 1000 live births^a^	Most frequent organ dysfunction	Most frequent cause of maternal near miss^b^	No. of maternal deaths	Maternal deaths per 100 000 live births^c^	Ratio of maternal near miss to maternal death^d^	Mortality index, %^e^
**Lower-middle-income countries**										
Ps et al., 2013^53^	India	7 330	131	17.9	NR	Haemorrhage	23	313	5.6	14.9
Tunçalp et al., 2013^36^	Ghana	3 206	94	28.6	Coagulation or haematological dysfunction	Severe postpartum haemorrhage	37	1 154	2.5	28.2
Kaur et al., 2014^43^	India	6 008	140	23.3	NR	Hypertensive disorders	16	266	8.8	10
Kushwah et al., 2014^48^	India	5 219	63	6.8	NR	Hypertensive disorders	47	901^f^	1.3	42.9
Luexay et al., 2014^61^	Lao People's Democratic Republic	1 123	11	9.8	Respiratory	Haemorrhage	2	179	5.5	15.3
Nacharajuh et al., 2014^55^	India	2 385	22	9.2	NR	Pre-eclampsia	2	84^f^	11.0	8.3
Pandey et al., 2014^42^	India	5 273	633	120.0	NR	Haemorrhage	247	45	2.6	27.2^f^
Bakshi et al., 2015^31^	India	688	51	74.1^f^	NR	Sepsis	10	1	5.1	16.4
Bashour et al., 2015^83^	Egypt	2641	32	12.1	Coagulation or haematological dysfunction	Haemorrhage	3	114	11.0	8.6
Mazhar et al., 2015^66^	Pakistan	12 729	94	7.0	Cardiovascular	Postpartum haemorrhage^g^	38	299	2.5	28.7
Sangeeta et al., 2015^27^	India	6 767	27	4.0	Coagulation or haematological dysfunction	Haemorrhage	13	188^f^	3.4	22.8
Abha et al., 2016^49^	India	13 895	211	15.2	Coagulation or haematological dysfunction	Hypertensive disorders	102	734^f^	2.1	32.9
Ansari et al., 2016^65^	Pakistan	1 035	76	73.4^f^	Cardiovascular	NR	7	676	10.9^f^	8.4^f^
De Mucio et al., 2016^30^	Honduras	613	10	16.3^f^	NR	NR	1	163	10.0	9.1^f^
De Mucio et al., 2016^30^	Nicaragua	477	4	8.4^f^	NR	NR	0	0	0	0
Kulkarni et al., 2016^50^	India	14 508	525	36.2	Coagulation or haematological dysfunction	Hypertensive disorders	NR	648^f^	5.6	9.6
Oladapo et al., 2016^62^	Nigeria	91 724	1451	15.8	NR	Obstetric haemorrhage	998	1 088	2.5^f^	40.8
Parmar et al., 2016^35^	India	1 929	40	20.7	NR	NR	2	933	2.2	31.0
Rathod et al., 2016^51^	India	22 092	167	7.6	Coagulation or haematological dysfunction	Haemorrhage	66	298	3.4	29.7
Ray et al., 2016^33^	India	4 038	218	54.0	NR	Hypertensive disorders	17	421	13.0	7.17
Tanimia et al., 2016^40^	Papua New Guinea	13 338	121	9.1	NR	Obstetric haemorrhage	9	67	13.5	6.8
Bolnga et al., 2017^68^	Papua New Guinea	6 019	153	25.4	NR	Postpartum haemorrhage	10	166	15.3	6.8
Chandak & Kedar, 2017^52^	India	12 757	137	10.7	Cardiovascular	Eclampsia	NR	243^f^	10.5	18.5^f^
Mbachu et al., 2017^28^	Nigeria	262	52	198.0	NR	Hypertensive disorders	5	1 908	11.4	8.8
Tallapureddy et al., 2017^56^	India	3 784	32	8.5	Coagulation or haematological dysfunction	Haemorrhage	6	159^f^	5.3	15.8
Oppong et al., 2019^47^	Ghana	8 433	288	34.2	Cardiovascular	Pre-eclampsia and eclampsia^h^	62	735	4.6^f^	21.7^f^
Panda et al., 2018^32^	India	1 349	89	66.0	NR	Severe pre-eclampsia	8	593	11.1	8.2
Reena & Radha, 2018^54^	India	3 451	32	9.3	Coagulation or haematological dysfunction	Severe pre-eclampsia	5	145	6.4	13.5^f^
Chaudhuri & Nath, 2019^57^	India	4 081	175	43.0	Vascular dysfunction	Hypertensive disorder (eclampsia)	23	564	7.7	11.5
Chhabra et al., 2019^58^	India	38 111	261	6.9	Coagulation	Hypertensive disorder	166	436	1.6	23
El Agwany, 2019^46^	Egypt	28 877	170	5.9	Coagulation	Haemorrhage	14	50^f^	12.2	7.5
Gabbur et al., 2019^59^	India	6 053^i^	100	16.4	NR	Postpartum haemorrhage	13	215^f^	7.7	88.5^f^
Herklots et al., 2019^69^	United Republic of Tanzania	22 011	256	11.6	Coagulation or haematological dysfunction	NR	79	359	3.2	24.0
Jayaratnam et al., 2019^71^	Timor-Leste	4 529	39	8.0	NR	Eclampsia or postpartum haemorrhage	30	662	1.3	43.0
Mansuri & Mall, 2019^60^	India	21 491	247	11.5	NR	Eclampsia or pre-eclampsia	79	367	3.1	24.2
Karim et al., 2020^67^	Pakistan	3 360	54	16.0	NR	Adherent placenta	8	238	6.8	12.9
Lilungulu et al., 2020^70^	United Republic of Tanzania	3 480	124	36.0	NR	Haemorrhage	16	460	7.8	11.4
Owolabi et al., 2020^22^	Kenya	36 162	260	7.2	NR	Postpartum haemorrhage	13	36	20.0	4.8
Samuels & Ocheke, 2020^64^	Nigeria	2 357	86	36.5^f^	NR	Hypertensive disorders	19	806	4.5	81.9^f^
Ugwu et al., 2020^63^	Nigeria	2 236^k^	60	26.8^f^	Cardiovascular	Severe haemorrhage	28	1251	2.1	31.8
**Upper-middle-income countries**										
Cecatti et al., 2011^24^	Brazil	14 418	194	13.5	NR	NR	18	125	10.7	8.5
Morse et al., 2011^23^	Brazil	1 069	10	9.4	NR	Severe pre-eclampsia^g^	3	280	3.3	23
Lotufo et al., 2012^25^	Brazil	9 683	43	4.4	NR	Haemorrhage	5	52	8.6	10.4
Jabir et al., 2013^82^	Iraq	25 472	129	5.1	Cardiovascular	Obstetric haemorrhage	16	63	9.0	11.0
Shen et al., 2013^26^	China	18 104	72	4.0	NR	Postpartum haemorrhage	3	16	23.0	4.2
Dias et al., 2014^72^	Brazil	23 894	243	10.2	NR	NR	7	29	34.7	2.8
Galvão et al., 2014^74^	Brazil	16 243	76	4.7	NR	Hypertensive disorders^j^	17	105	4.5	18
Bashour et al., 2015^83^	Lebanon	1 171	5	4.3	Hepatic dysfunction	Multiple causes^k^	0	0	0	NR
Madeiro et al., 2015^75^	Brazil	5 841	56	9.6	NR	Hypertensive disorders	10	171	5.6	15.2
Naderi et al., 2015^80^	Islamic Republic of Iran	19 908	501	25.2	NR	Severe pre-eclampsia	2	10^f^	250.0	NR
Oliveira & Da Costa, 2015^76^	Brazil	19 940	255	12.8	NR	Hypertensive disorders	NR	280^f^	4.5	18
Soma-Pillay et al., 2015^37^	South Africa	26 614^i^	114	4.3^f^	Vascular	Obstetric haemorrhage	NR	71^f^	7.1^f^	14
Cecatti et al., 2016^73^	Brazil	82 144	770	9.37	NR	Hypertensive disorders	140	170	5.5	15.4
De Mucio et al., 2016^30^	Colombia	334	3	9.0^f^	NR	NR	0	0	0	0
De Mucio et al., 2016^30^	Dominican Republic	133	3	22.6^f^	NR	NR	0	0	0	0
De Mucio et al., 2016^30^	Ecuador	228	2	8.9^f^	NR	NR	0	0	0	0
De Mucio et al., 2016^30^	Paraguay	334	2	6.0^f^	NR	NR	1	299^f^	2.0^f^	33.3^f^
De Mucio et al., 2016^30^	Peru	315	11	35.0^f^	NR	NR	0	0	0	0
Ghazivakili et al., 2016^81^	Islamic Republic of Iran	38 663	192	5.0	Cardiovascular	Severe pre-eclampsia	NR	18^f^	2.4	3.5
Mohammadi et al., 2016^39^	Islamic Republic of Iran	12 965	82	6.3	Coagulation or haematological dysfunction	Severe postpartum haemorrhage	NR	93^f^	6.9^f^	13
Norhayati et al., 2016^29^	Malaysia	21 579	47	2.2	Coagulation or haematological dysfunction	Postpartum haemorrhage	NR	9^f^	23.5	4.1
Akrawi et al., 2017^41^	Iraq	17 353	142	8.2^f^	Cardiovascular	Hypertensive disorders	11	63	12.9	7.2
Iwuh et al., 2018^44^	South Africa	19 222	112	5.8	NR	Hypertensive disorders	13	68	8.6	10.4
Oliveira Neto et al., 2018^77^	Brazil	8 065	60	7.4	Hepatic dysfunction	Pre-eclampsia	NR	62^f^	13.0	7.7^f^
De Lima et al., 2019^34^	Brazil	1 002	55	54.8	Respiratory	Hypertension	1	99	11.0	8.3
Mu et al., 2019^79^	China	9 051 638^l^	37 060	4.1^f^	Coagulation dysfunction	Hypertensive disorders	380	4.1^f^	97.5	NR
Heemelaar et al., 2020^38^	Namibia	37 106	298	8.0	NR	Obstetric haemorrhage	23	62	13.0	92.8^f^
Ma et al., 2020^78^	China	542 109	3208	5.9	Coagulation or haematological dysfunction	Postpartum haemorrhage	34	6.3	94.4^f^	1.1
Verschueren et al., 2020^45^	Suriname	9 114	71	7.8	Coagulation or haematological dysfunction	Hypertensive disorders	10	110	7.1^f^	12.0

NR: not reported.

^a^ Maternal near miss ratio.

^b^ Most frequent causes of maternal near miss; terminology as used in the original article.

^c^ Maternal mortality ratio.

^d^ Ratio of number of maternal misses to the number of maternal deaths.

^e^ Mortality index is: [number of maternal deaths / (number of cases of maternal near miss + number of maternal deaths) × 100].

^f^ We calculated the value shown using formulae shown in the main text.

^g^ Severe maternal outcome.

^h^ Potentially life-threatening conditions.

^i^ Per number of births.

^j^ Severe acute maternal morbidity.

^k^ Multiple causes: placenta praevia, placenta accreta, placenta increta, placenta percreta, hepatic disease.

^l^ Number of pregnant women.

Studies reported a total of 2917 maternal deaths. The median maternal mortality ratio for all middle-income countries was 163 per 100 000 live births (IQR: 52–367), with a median of 306 per 100 000 live births (IQR: 162–666) in lower-middle-income countries versus 62 per 100 000 live births (IQR: 9–105) in upper-middle-income countries. The median mortality index in middle-income countries was 13.5% (IQR: 8.4–24.0%), ranging from 15.8% (IQR: 9.0–28.5%) for lower-middle-income countries to 10.7% (IQR: 7.3–15.4%) for upper-middle-income countries. 

### Causes

Hypertensive disorders of pregnancy and obstetric haemorrhage were the commonest causes of maternal near miss. In the lower-middle-income countries, the most frequent cause of near misses was haemorrhage (including reported severe postpartum haemorrhage, obstetric haemorrhage, postpartum haemorrhage, haemorrhage and placenta praevia), reported in 18 out of 40 studies (45%) from 10 countries. Hypertensive disorders of pregnancy (including severe pre-eclampsia and eclampsia) were the cause of near miss in 15 studies (38%) from four countries. In the upper-middle-income countries, hypertensive disorders of pregnancy were the commonest cause of maternal near miss in 15 out of 29 studies (52%) from six countries. Obstetric haemorrhage was reported as the commonest cause in eight studies (29%) from seven countries. In both lower-middle- and upper-middle-income countries, the main identified organ failure was coagulation or haematological dysfunction (which included haemorrhage with a minimum of 5 units of blood for transfusion and a platelet count < 50 000 platelets/mL). Cardiovascular organ dysfunction (shock, cardiac arrest) was the second most common organ failure.

### Adaptations

Adaptations to the maternal near-miss tool were suggested in 33 out of 69 (48%) studies. These modifications and difficulties in applying the WHO maternal near-miss tool are described in Table 3. Seven studies recommended reducing the threshold for defining major haemorrhage from 5 units of blood required for transfusion to 4 units,^38^^,^^39^ 3 units^30^^,^^40^^,^^41^ or even 2 units,^22^^,^^42^ to account for limited availability of blood. Other additions to the maternal near-miss tool suggested by researchers were: a definition of shock and sepsis (obstetric and non-obstetric); estimation of blood loss; bedside clotting time; severe anaemia; use of vasoactive drugs; assessing keto-acids in urine; and application of an oxygen face mask. In five studies, researchers recommended inclusion of admission to an intensive care unit as a criterion.^32^^,^^34^^,^^40^^,^^41^^,^^43^ Moreover, additional diagnoses to the current six life-threatening conditions criteria were advised, such as: placental abruption; medical and surgical disorders; diabetic keto-acidosis; acute collapse or thromboembolism; and non-pregnancy-related infections.^37^^,^^38^^,^^44^^,^^45^

**Table 3:** Difficulties reported and modifications applied to the World Health Organization maternal near-miss tool in middle-income countries

Author	Setting	Modifications applied in study	Comments and problems reported by study researchers
**Lower-middle-income countries**			
Kaur et al., 2014^43^	India, Himachal Pradesh	Addition of items to clinical criteria (severe pre-eclampsia; eclampsia)^a^ Addition of item to laboratory criteria (sepsis)^b^ Addition of item to management criteria (intensive care unit admission)	NA
Kushwah et al., 2014^48^	India, Madhya Pradesh	NA	Maximum units of blood available in study institute were 3 units as blood bank was not well supplied. Researchers believed that WHO’s criterion of receiving 5 or more units of blood was less applicable in a resource-poor institute.
Luexay et al., 2014^61^	Lao People's Democratic Republic	Simplified modification of WHO tool for use in the community^c^	Researchers concluded that maternal near misses could have been underestimated by application of the WHO definition of maternal near miss, which relies on good laboratory and management-based criteria. Adaptation of near-miss criteria for low-resource settings may benefit lower-middle-income countries where health services are also poorly resourced.
Pandey et al., 2014^42^	India	Omission of markers from laboratory criteria (pH; PaO_2_/FiO_2_) Lowering threshold for use of blood products to 2 units of blood	NA
Sangeeta et al., 2015^27^	India	NA	Researchers concluded that in low-resource settings, interventions need to be developed with the local context in mind.
Kulkarni et al., 2016^50^	India, Maharashtra	Addition of item to clinical criteria (anaemia)^d^	NA
Parmar et al., 2016^35^	Papua New Guinea	Omission of markers from laboratory criteria (pH; lactate; glucose and keto-acids in urine; PaO_2_/FiO_2_) Lowering threshold for use of blood products to 3 units of blood Addition of criteria (continuous use of vasoactive drugs; intensive care unit admission)	Data collection in accordance with WHO maternal near-miss guidelines, adjusted for local factors, is possible in a busy maternity unit in a resource-poor setting. Researchers concluded that such data have the potential to improve early detection of life-threatening conditions and hence obstetric outcomes.
Parmar et al., 2016^35^	India	NA	Researchers noted that the WHO classification was remarkable for identifying the most serious cases with higher risk of death. However, the WHO classification showed a high threshold for detection of maternal near miss. Researchers therefore concluded that the method was missing a significant proportion of women with conditions such as pre-eclampsia and eclampsia.
Bolnga et al., 2017^68^	Papua New Guinea	NA	Papua New Guinea’s resource-poor setting lacks the capacity to perform some of the WHO-recommended laboratory investigations such as pH and lactate. Researchers noted that use of locally relevant criteria was also important to avoid underestimation of the true burden of maternal near miss as previously reported in other resource-poor settings.
Panda et al., 2018^32^	India, Odisha	Addition of items to clinical criteria (haemorrhage; hypertensive disorders; abortion; sepsis) Addition of items to management criteria (intensive care unit admission) Addition of definitions of critical interventions (emergency postpartum hysterectomy; immediate blood transfusion)	NA
El Agwany, 2019^46^	Egypt	NA	Researchers could not apply the criteria due to lack of resources.
Gabbur et al., 2019^59^	India, Karnataka	NA	Researchers concluded that modification of the WHO tool is required as currently it leads to underestimation of maternal near miss.
Herklots et al., 2019^69^	United Republic of Tanzania, Zanzibar	Not modified (researchers reported the tool was applicable in this setting)	Conclusions about maternal near miss are dependent on the quality of data and challenges to this should be acknowledged. Researchers recommended adhering to the WHO criteria (adjusted to specific settings as needed) to enable meaningful comparison between similar reference populations.
Jayaratnam et al., 2019^71^	Timor-Leste	Not modified	Determining a clear diagnosis in a woman with maternal near miss is difficult due to presence of multiple symptoms, lack of diagnostics due to fast deterioration of the woman and lack of laboratory-based markers. Researchers concluded that maternal near-miss criteria must be modified to the local context to enhance incorporation of cases (e.g. requiring lower transfusion requirements) in future studies.
Oppong et al., 2019^47^	Ghana	Addition to definition of coagulation in organ dysfunction criteria (bedside clotting time of > 7 mins)	Organ system-based criteria are regarded as the most specific means of identifying maternal near miss. However, researchers argued that these criteria require ready availability of laboratory tests and medical technologies, thus impeding their use in many low-resource local settings.
Owolabi et al., 2020^22^	Kenya	Adjustments were: lowering threshold for use of blood products to 2 units of blood (Kenyan method) Addition of items (laparotomy; definition of shock; treatment with oxygen face mask)	Kenyan method yielded 1.4 times the numbers of maternal near miss than the WHO method. Researchers concluded that there is under-reporting using the WHO maternal near-miss method.
**Upper middle-income countries**			
Morse et al., 2011^23^	Brazil, Rio de Janeiro	NA	As bed availability and intensive care unit admission criteria are not the same, researchers noted that use of intensive care unit admission as a marker is questionable because it is affected by level of complexity of care in a health setting and organization of obstetric care.
Lotufo et al., 2012^25^	Brazil, São Paulo State	NA	Researchers reported no difficulties in using and identifying the WHO criteria, with the exception of certain clinical criteria (e.g. gasping, cyanosis and bedside clotting tests) which generally occurred before starting complex care in the intensive care unit.
Shen et al., 2013^26^	China	NA	The study applied 16 of the 25 WHO criteria. Researchers noted that some women in their study received blood transfusion of < 5 units or intubation related to anaesthesia and therefore did not meet the WHO criteria. Women with pre-eclampsia without jaundice and loss of consciousness for < 12 hours were not included in the WHO clinical criteria group. In the laboratory-based group, women with maternal near miss were differentiated by oxygen saturation, blood creatinine level, platelet count and total bilirubin. Researchers reported it was impossible to always obtain blood pH or lactate level, because these parameters were not routinely checked in their institute.
Naderi et al., 2015^80^	Islamic Republic of Iran	Beside the collection of data on life-threatening disease, researchers added a form based on a published method.^5^ Four groups were added to the form (haemorrhagic; hypertensive; management; and systemic disorders)	NA
Oliveira & Da Costa, 2015^76^	Brazil, Pernambuco	NA	Mechanical ventilation was required in less than one quarter of cases of maternal near miss. Researchers noted that this finding may be attributed to local differences in accessibility of resources and interventions. It is one of the drawbacks of criteria based only on treatment because a more complex hospital and laboratory structure is required.
Soma-Pillay et al., 2015^37^	South Africa	NA	The WHO tool identified five potentially life-threatening conditions: severe postpartum haemorrhage; severe pre-eclampsia; eclampsia; sepsis or severe infection; and ruptured uterus. Researchers noted that conditions such as abruptio placentae, non-obstetric infections and medical and surgical disorders were also important causes of maternal morbidity. Researchers recommended that the WHO tool should expand the categories of potentially life-threatening conditions.
Ghazivakili et al., 2016^81^	Islamic Republic of Iran	NA	Researchers noted that a limitation of the WHO tool is that application of criteria based on organ failure requires relatively sophisticated laboratory and clinical monitoring. Underestimating occurrence of maternal near miss due to lack of equipment or unavailability of some tests is therefore possible.
Mohammadi et al., 2016^39^	Islamic Republic of Iran	Lowering threshold for use of blood products to 4 units of blood Increasing threshold for platelets to < 75 000 per mL Addition of items to laboratory criteria (rapid reduction of > 4 g/dL in haemoglobin concentration)	NA
Norhayati et al., 2016^29^	Malaysia	NA	Researchers noted that use of the WHO criteria was limited in smaller health facilities. Laboratory-based markers (e.g. pH, PaO_2_, lactate) and management-based markers (e.g. vasoactive drugs and hysterectomy) were less likely to be applicable in these health facilities.
Akrawi et al., 2017^41^	Iraq	Lowering threshold for use of blood products to 3 units of blood Addition of item to management criteria (admission to close observation care unit > 6 hours) Addition of items to clinical criteria (prolonged labour;^e^ anaemia)^f^	NA
Iwuh et al., 2018^44^	South Africa	Addition of items to definition of severe maternal complications (acute collapse or thromboembolism; non-pregnancy-related infections; medical or surgical disorders)	NA
Oliveira Neto et al., 2018^77^	Brazil, São Paulo State	NA	Researchers noted that arterial blood gas sampling was not routinely collected in all pregnant or postpartum patients admitted to the intensive care unit. PaO_2_ records were missing in some cases of maternal near miss. When evaluation of the level of consciousness by the Glasgow coma scale was compromised (due to residual effects of anaesthetics in the postoperative period, or by the use of continuous sedation), the Glasgow coma score of 15 was used as a criterion. Management criteria and not laboratory criteria would be useful to identify severe maternal outcome because they are more related to organ failure. Researchers noted that arterial blood gas sampling was not routinely collected in all pregnant or postpartum patients admitted to the intensive care unit. PaO_2_ records were missing in some cases of maternal near miss. When evaluation of the level of consciousness by the Glasgow coma scale was compromised (due to residual effects of anaesthetics in the postoperative period, or by the use of continuous sedation), the Glasgow coma score of 15 was used as a criterion. For the variable use of vasoactive drugs, researchers noted that WHO does not establish any other criteria for stratification of severity (e.g. blood pressure levels or whether vasodilator or vasoconstrictor drug used) which could be useful for this purpose. Researchers argue that these issues should be better addressed and possibly changed.
De Lima et al., 2019^34^	Brazil, Alagoas	Researchers noted that intensive care unit admission was not included in the WHO criteria but was an important marker of maternal severity in their study (identified in 94.5% of pregnant women)	Researchers noted that, in contrast to laboratory and management criteria, clinical criteria are important for low-income regions, because no complex laboratory and hospital infrastructures are required. Limitations of laboratory and management criteria are that most of these criteria require high-complexity units, wards, equipment or facilities for their use. Women experiencing near miss may therefore be missed. Lowering the numbers of packed red blood cell units or including disease-based criteria was necessary in low-resource settings to classify women as near miss.
Mu et al., 2019^79^	China	NA	Lack of high-quality medical institutions in rural areas is a problem for maternal health. In recent years, China has strengthened management of women with severe complications so that they must give birth in tertiary hospitals. The researchers argued that the lack of tertiary hospitals in rural areas will affect accessibility of pregnant women to high-quality health care.
Heemelaar et al., 2020^38^	Namibia	Adapted tool for middle-income countries Lowering threshold for use of blood products to 4 units of blood Addition of criteria (laparotomy other than caesarean section or ectopic; pregnancy < 12 weeks) Addition of items to clinical criteria (eclampsia; uterine rupture; non-obstetric sepsis)	The researchers noted the limited availability of laboratory tests and management options resulting in under-reporting of maternal near miss.
Verschueren et al., 2020^45^	Suriname	Evaluation of the WHO maternal near-miss tool by comparing the Suriname obstetric surveillance system with WHO maternal near miss, Namibian and sub-Saharan African tools, to identify the most useful method	The researchers concluded that the WHO tool leads to underestimation of the prevalence of severe complications as the tool does not include certain disease-based conditions.
**Multiple countries**			
De Mucio et al., 2016^30^	Colombia, Dominican Republic, Ecuador, Honduras, Nicaragua, Paraguay, Peru	Omission of items from laboratory criteria (glucose and keto-acids in urine) Lowering the threshold for use of blood products to 3 units of blood	NA

NA: not applicable; PaO_2_: oxygen arterial pressure; PaO_2_/FiO_2_: ratio of arterial oxygen partial pressure to fractional inspired oxygen**;** WHO: World Health Organization.

^a^ Severe pre-eclampsia (blood pressure of 170/110 mmHg measured twice); proteinuria of 5 g or more in 24 hours; and HELLP syndrome (haemolysis, elevated liver enzymes and low platelets) or pulmonary oedema or jaundice or eclampsia (generalized fits without previous history of epilepsy) or uncontrollable fits due to any other reason.

^b^ Sepsis or severe systemic infection, fever (> 38 °C), confirmed or suspected infection (e.g. chorioamnionitis, septic abortion, endometritis, pneumonia), and at least one of the following: heart rate > 90 beats per minute, respiration rate > 20 breaths per minute, leukopenia (white blood cells < 4000/μL), leukocytosis (white blood cells > 12 000/μL).

^c^ See the supplementary files of the original article for the complete list.^61^

^d^ Anaemia was defined by the researchers as haemoglobin level of < 60 g/L or clinical signs of severe anaemia without acute haemorrhage.

^e^ Abnormal or difficult childbirth or labour for more than 24 hours.

^f^ Low haemoglobin level (< 6 g/dL) or clinical signs of severe anaemia in women without severe haemorrhage.

Note: See Box 1 for the WHO inclusion criteria.

Some studies reported problems with applying the tool, including underestimation of maternal near miss by using only criteria based on organ dysfunction;^35^^,^^84^ and difficulties with identifying women with near miss because the necessary equipment and facilities were unavailabile^14^ or due to time pressure in clinical emergencies.^36^ Researchers also reported that difficulties with categorization of the WHO maternal near-miss criteria and different interpretations of the tool would make comparisons problematic.^37^

## Discussion

The WHO maternal near-miss tool facilitated evaluation of the maternal near-miss ratio in 26 middle-income countries. The main reported causes of maternal near miss were hypertensive disorders in pregnancy and obstetric haemorrhage. The maternal near-miss ratios were considerably higher in lower-middle- than upper-middle-income countries (median: 15.9 versus 7.8 per 1000 live births). This finding is not unexpected due to differences in countries’ resources, but is an important finding about the validity of the maternal near-miss approach. Lower-middle-income countries also had considerably higher maternal mortality ratios and mortality indices than upper-middle-income countries.

The median maternal near-miss ratios per 1000 live births in middle-income countries in our study were higher than those in previous studies of high-income countries (for example, 1.8 in Ireland and 2.0 in Italy)^85^^,^^86^ and lower than those in low-income countries (for example, 17.0 in Ethiopia, 88.6 in Somalia and 23.6 in United Republic of Tanzania).^15^^,^^87^^,^^88^ These differences might in part be explained by differences in quality of care, reflected by the mortality index, where the higher the index, the more women with life-threatening conditions die. Comparisons of maternal near-miss ratios and sharing lessons learnt from audits in different regions or countries might benefit maternal health worldwide.

Monitoring maternal near misses and maternal deaths showed differences not only among middle-income countries but also across different settings of the same countries. Differences between rich and poor or urban versus rural populations are often large in middle-income countries. Outcomes will differ depending on the quality of care and socioeconomic circumstances in different regions.^19^^,^^20^

Adaptations to the WHO maternal near-miss tool have previously been considered for high- and low-income countries.^14^^–^^16^ We found that various adaptations of the WHO tool were also suggested by researchers in middle-income countries, depending on the setting. Adaptation of the tool hampers comparisons across different settings, but may sometimes be necessary to prevent under-reporting of severe morbidity. Several of the included studies recommended reducing the threshold for defining major haemorrhage, or making additions to the WHO criteria. Researchers in our study mentioned the limitations of under-reporting maternal near miss using the current WHO criteria based on organ dysfunction. These limitations, however, have also been reported in both low- and high-income countries.^11^^,^^13^^,^^15^^,^^16^ While some studies limited the organ-dysfunction criteria only to life-threatening conditions, other studies added up to six diagnoses of severe maternal complications or critical interventions from the list of WHO criteria in Box 1. Moreover, in the original search, we had to exclude 39 studies applying different criteria that were too far from the original WHO criteria and seven studies whose criteria were unclear.

The issues mentioned above show that the maternal near-miss tool is helpful in recognizing severe morbidity, but may benefit from adaptations to be locally applicable. The major aim of the tool is that lessons for clinical care are drawn. Only including cases of maternal near miss that occur in tertiary level hospitals does not provide a comprehensive picture of maternal near miss in a country. Especially in middle-income countries, differences in quality of care in facilities are large between richer and poorer populations, those living in urban versus rural areas and those using public versus private facilities.^89^ The WHO criteria can be seen as a package of minimum criteria that should be in place to provide appropriate care. These minimum criteria may create an incentive for countries to upgrade their diagnostic and therapeutic capacity to improve health equity.

A limitation of our study is that small differences in methods of identification of maternal near miss between countries could result in major differences in outcomes. Moreover, we had to exclude a considerable proportion of studies that used different criteria to identify maternal near miss. This underlines the complexity of the challenge when aiming to compare maternal near miss across different countries and settings. An additional list of diagnoses would be a valuable contribution to reflect actual health problems in different settings.^37^^,^^38^^,^^44^^,^^45^ This issue was also discussed in a study published by our team after this search in 2021.^90^ Our search was performed without any language restriction and in large databases, but it is still possible that the search may have missed studies.

A strength of our study was the relatively large number of publications that allowed us to obtain a comprehensive overview of maternal near miss in middle-income countries and to make robust comparisons between different regions and countries. We only report data about maternal near miss from 26 of the world’s 105 middle-income countries. We excluded some studies of near miss from our review because they used different criteria from the WHO near-miss criteria or did not clearly report the criteria used. Nevertheless, the countries analysed here reported large numbers of live births as denominator populations, providing a relatively robust and comprehensive overview of maternal near-miss ratios. We found multiple studies for Brazil and India, with India showing a particularly broad range of outcomes. These data for India reflect the large differences within this large country, indicating that smaller studies might not be representative for the entire territory.^31^^–^^34^

We conclude that instead of adapting the WHO maternal near-miss tool, the foremost important aim of the tool should be to improve the quality of maternity care from lessons learnt by performing audits of cases of maternal near miss.
